# Systematic review in relation to support of diversity in nursing homes

**DOI:** 10.3389/fragi.2024.1389610

**Published:** 2024-09-24

**Authors:** Javier Mesas-Fernández, Jordi Tous-Pallarès, Ivette Margarita Espinoza-Díaz

**Affiliations:** ^1^ Faculty of Health, Valencian International University, Castelló de la Plana, Spain; ^2^ Department of Psychology, University of Rovira i Virgili, Tarragona, Spain

**Keywords:** diversity, nursing homes, elderly, person-centered care model (PCCM), discrimination

## Abstract

**Introduction:**

Given the increasing global population of older adults, it is essential and inevitable that healthcare centers and nursing homes address and accommodate diversity in their support systems as interventions for healthy aging. The active aging and the inclusion of all people regardless of their religion, origin, and/or sexual orientation is essential to create a climate of safety. Discrimination must be addressed from all angles, at the social level, at the business level and by all workers in nursing homes.

**Methodology and results:**

This study provides a comprehensive review of existing literature to systematize information on diversity among older adults in healthcare centers and nursing homes. Out of 1.458 articles identified, 10 were analyzed in depth, revealing that addressing diversity among older adults is crucial to overall mental and physical healthy aging. The findings underscore the need for a multidisciplinary approach and effective management through the Person-Centered Care Model (PCCM).

**Discussion and conclusion:**

This study highlights the critical role of the Person-Centered Care Model (PCCM) in addressing diversity in aging needs. It highlights the necessity of tailoring care based on individual life histories and experiences. Additionally, it calls for the implementation of inclusive policies in nursing homes and emphasizes the need for professional training on diversity to ensure these facilities are safe and supportive for all residents.

## 1 Introduction

It is necessary to offer a more inclusive concept about aging, which accepts and adopts diversity in all its forms, considering the biological, psychological, and social aspects of the person, and not only from the economic point of view. It is important to expand the active aging model. This action must go hand in hand with an effort to carry out actions and activities where the differences of each human being are highlighted and enhanced (Villar Possada et al., 2018).

Diversity in aging is a phenomenon that needs to be dealth with and discussed since it poses a series of challenges that require attention in the new realities within nursing homes. The importance of studying the phenomenon from a comprehensive vision is highlighted due to the challenges, opportunities, transformations, and concerns that this phenomenon represents in society.

According to Sánchez Barrera et al. (2019), the aging process is surrounded by a range of beliefs, myths, prejudices, policies, and perceptions, emphasizing the need for a more in-depth exploration of this topic. The increasing global population of older adults is viewed as a social achievement due to longer life expectancy. However, this also presents a significant challenge: transforming the concept of healthy aging from a rare privilege into an opportunity that embraces all forms of diversity. The challenge is that of healthy aging, passing from the belief that it is an exception and a privilege, including all types of diversities, to be an opportunity. Support for diversity is a necessity. The inclusion of all people regardless of their religion, origin, and/or affective-sexual diversity is essential to create a climate of safety.

In relation to older people, diversity is a key concept to understand and adequately address the needs and realities of this population. Diversity refers to the approach to equality that seeks to celebrate the differences between people (Klein, 2016). Older people are not a homogeneous group but present a great variety in terms of their characteristics and needs. Some are active and healthy, while others may have functional and/or cognitive diversity. In addition, older people also have differences among themselves in terms of culture, wealth, social status, gender, affective, sexual, etc. In the same way as the rest of the population.

Diversity refers to the presence of people with different physical, social, and personal characteristics in a group or organization. These characteristics include race, ethnicity, age, gender, sexual identity, religion, physical and mental ability, language, income, and education. One’s concept of diversity is linked to one’s experiences of diversity, one’s own social, political, educational values and perspectives, etc. (Nash, 2001).

Sexuality in human beings is something innate. There is no limit to this dimension since it is part of our communication. On many occasions, and mistakenly, sexuality is related to sexual activity itself. Sexuality as a dimension covers aspects such as identity, intimacy, attitudes, gender roles, sexual orientation, desire, etc. In relation to the affective-sexual diversity, on the other hand, refers to the different ways of expressing affection, desire, and erotic and/or loving practices. That is, to all the possibilities of accepting, assuming and living affectivity and sexuality, whether for people of the same sex, the opposite sex or both (Morales-Rodríguez, 2021).

According to Boyon (2021), through the Ipsos report carried out in 2021 on 19,069 people, various issues regarding the LGTBI + elderly group are made clear:

- By the year 2050, it is expected that there will be about 2,000 million older people throughout the planet; 22% of the planet’s inhabitants will be over 65 years old by that date. It is estimated that, of those 2,000 million people, approximately, 200 million will be LGTBI+.
- Spain is the third country in the world, the first in Europe, with the highest percentage of the population that declares themselves non-heterosexual (12% in total; of which 6% say they are bisexual, 5% gay and the other 1% pansexual).

The lack of specific data can make it difficult to articulate different public policies aimed at improving the lives of older people. Due to this, as Equala (2023) indicates, in recent years an incipient effort has been made to generate indicators that allow us to understand the reality of LGTBI + people.

In relation to the problem of racism and religion, in society, this could create two fundamental aspects that need to be considered. Firstly, the importance of religion in older people and, consequently, in nursing homes. Secondly, the possibility of racist attitudes being replicated in the centers by users and staff. For Kaplan and Berkman (2022), there is a relationship between the concepts of religion and spirituality, which, although they are similar, are not identical. They refer to religion as a more formal and structured concept, and includes more traditional activities, the performance of rituals and putting them into practice. On the other hand, when they talk about spirituality, they do so by mentioning the immaterial and intangible, which does not have to be related to a specific group.

According to Farrell et al. (2022) constructs of racism and ageism can have negative effects on health outcomes that can be magnified when race and age intersect, including recent events such as the COVID-19 pandemic.

The Person-Centered Care Model (PCCM) proposes an approach considering the care of the person, their dignity and their wishes, in addition, it pays special attention to their decisions, tastes and preferences, understanding that each person has a unique identity that is differentiated from the rest.

All of this with the objective that every older person, regardless of their dependency, develops their own vital process. Maintaining dignity throughout this process is essential to promoting their quality of life.

Care encompasses those individual and social practices designed to ensure the survival and wellbeing of human beings. Caring means supporting people in their autonomy and helping them to function in daily life (HelpAge Spain International, 2021). People who need care have the right to exercise control directly or indirectly over their lives and care. The need for new forms of care that focus on putting the person at the center is becoming evident.

The diversity of the older population is a key factor in providing services and care tailored to the individual needs of each person. PCCM is particularly relevant in this sense, as it promotes the personalization and adaptation of services and care to the individual needs and preferences of each person.

Population aging is a global phenomenon, with middle-income and low-income countries experiencing the most rapid growth in individuals over 60, increasing from 16% in 2019 to 36% in recent years (World Health Organization, 2021). This trend highlights the pressing issue of ageism, which manifests in societal stereotypes, prejudices, and discriminatory practices (World Health Organization, 2021). As the older adult population continues to rise, the demand for healthcare centers and nursing homes will increase, necessitating a focus on diversity as a foundational element. It is imperative to ensure that individuals with diverse characteristics and needs have access to appropriate resources and environments that promote their wellbeing. Embracing diversity and implementing individualized care models are crucial for enhancing nursing home services. A comprehensive review of management practices, including attention to internal policies, protocols, records, and diversity training for professionals, is essential for improving care in these settings.

This article contributes significantly to the field of research by highlighting the need for a more inclusive concept of ageing, which addresses diversity in all its forms and considers the biological, psychological and social aspects of older people. Research indicates that ageing should be an accessible opportunity for all, regardless of their affective-sexual, cultural, religious or socioeconomic diversity.

## 2 Methodology

This systematic review is based on the Preferred Reporting Items for Systematic Reviews and Meta-Analyses (PRISMA). The PICO framework is used to structure the systematic review approach and is related to diversity in older adults living in healthcare facilities and nursing homes. A non-experimental design was utilized to perform a systematic review of the literature concerning diversity in older adults living in nursing homes. This method involved aggregating and analyzing contributions from various peer-reviewed literatures to determine current levels of support for diversity in nursing homes. This analysis was also used to inform best interventional practices to provide nursing home residents with a supportive healthy environment centered around PCCM.

Carrying out systematic research requires the establishment of three phases, these are developed and articulated sequentially to guarantee the identification and selection of documents (Table 1).

**TABLE 1:** Phases to guarantee the identification and selection of documents.

Phase	Step	Description
Identification	1. Literature Search	Conduct comprehensive searches in multiple databases to identify all relevant studies
	2. Database Management	Use reference management software to organize search results and remove duplicates
Screening	3. Title and Abstract Screening	Review titles and abstracts to exclude studies that do not meet the inclusion criteria
	4. Full-Text Retrieval	Obtain full-text copies of studies that appear to meet the inclusion criteria or for which there is insufficient information
Eligibility	5. Full-Text Screening	Assess the full-text articles for eligibility based on predefined inclusion and exclusion criteria Peer-reviewed literature
	6. Reasons for Exclusion	Document reasons for excluding studies after full-text review
Inclusion	7. Data Extraction	Extract relevant data from the included studies using a standardized form
	8. Quality Assessment	Assess the risk of bias and the methodological quality of the included studies
	9. Data Synthesis	Synthesize the data from the included studies, which may include a meta-analysis if appropriate

### 2.1 PICO question

The work developed and the methodology presented below are based on the question in relation to the PICO question, which is posed in the following table (Table 2). Table 2 details the PICO question, which outlines the objectives of this study.

**TABLE 2:** PICO question.

P	Patient, population or problem	Older people who may belong to diverse groups living in heatlh care facilities and nursing homes
I	Intervention or exposure	Suggestions for implementation of tailored diversity interventions within nursing homes and healthcare centers, including strategies to support underrepresented groups and training for staff on attitudes towards these groups
C	Comparison	Standard care practices without specific diversity-focused interventions
O	Outcome	Improvement in the quality of life for residents, including enhanced satisfaction and wellbeing, and the effectiveness of different interventional methods in supporting diversity

This table is transferred to the definition of the following PICO question:

How does attention to diversity influence the quality of life in older people?

### 2.2 Study selection criteria

- Inclusion criteria:
  • Scientific articles and magazines published in the last 5 years.
  • Publications in which the active aging approach, its components, policies, and strategies have been addressed, both in Spanish and English.
  • Articles under a qualitative design
  • Scientific documents and research that are complete.
  • Texts that have been developed from national and international cases.
  • Scientific articles and magazines, both national and international.
- Exclusion criteria:
  • Type of study: meta-analysis and systematic reviews; opinion articles and duplicates.
  • Sample population (age <60).
  • Documents that do not contain keywords in the title, summary and/or keywords.

Consequently, it was determined through the checklist that through the PRISMA 2020 declaration, that the research sources must contain specific sections such as: section/topic; qualification; summary; introduction; methods of inquiry and the resolutions that other researchers managed to achieve in their investigative process and that were contrasted in the discussion section.

#### 2.2.1 Information sources and search method

The following databases were searched: Pubmed (US National Library of Medicine), Cochrane Library, Scielo, Web of Science (Institute of Scientific Information) and Scopus.

For the search, different keywords were combined:

- Terms Mesh: quality of life, nursing homes, intersectionality.
- Terms DeCs: calidad de vida, residencias de personas mayores e interseccionalidad.

In addition, terms that are not included in the Mesh terms were used, they were the following: diversity, gerontology, and elderly people. These terms were combined with the Boolean operators “AND” and “OR”, in this way the search is directed to the desired articles. Finally, the following search is carried out in the different databases:

(religious OR spiritual OR cultural OR gender OR sexual) AND diversity AND (“quality of life” OR “wellbeing” OR “wellbeing” OR care) AND (“elderly people” OR “nursing homes” OR elder OR “old people”).

The present systematic review has been registered in PROSPERO with the following number: 543543 - Systematic review in relation to diversity in nursing homes.

Of all the articles obtained in the searches, a total of 1,458 articles; duplicate articles in the different databases and systematic reviews were discarded. A total of 1,311 articles were discarded, so 147 articles were selected for a first reading of the title and summary.

#### 2.2.2 Search list

The search in the databases was carried out between the months of May and July 2023, with the last search being carried out on 23 July 2023.

#### 2.2.3 Web of science

- The search was carried out within “Topic,” with the following combination:
- (religious OR spiritual OR cultural OR gender OR sexual) AND diversity AND (“quality of life” OR “wellbeing” OR “wellbeing” OR care) AND (“elderly people” OR “nursing homes” OR elder OR “old people”) AND “intersectionality”.
- A total of 598 articles were obtained. The filters were introduced: Spanish and English language, complete and open availability, and publication date between 2013 and 2023. The following “Meso topics” were used: 6.178 (Gender & Sexuality), 1.14 (Nursing), 6.73 (Social Phsycology), 1.155 (Medical Ethics), 6.86 (Human Geography), 1.66 (Hiv), 6.256 (Religion) 6.24 (Psychiatry & Psychology). A total of 45 articles were obtained for reading the title and abstract.

#### 2.2.4 Scopus

- The search was carried out within “Article title, abstract, keywords” with the following combination:
- TITLE-ABS-KEY (religious OR spiritual OR cultural OR gender OR sexual) AND diversity AND (“quality of life” OR “wellbeing” OR wellbeing” OR care)AND “elderly people” OR “nursing homes” OR elder OR “old people” AND “intersectionality”
- A total of 266 articles were obtained. Filters were introduced: Spanish and English language; publication date between 2019 and 2023; availability of the complete free open document; document type: article; In the subject area, documents related to Medicine, Nursing, Social Sciences, Arts and Humanities and Multidisciplinary were filtered, discarding the areas of Agriculture and Biological Sciences, Environmental Sciences and Neuroscience. A total of 22 articles were obtained, selected for reading the title and abstract.

#### 2.2.5 Pubmed

- The search was carried out within “Title/abstract” with the following combination:
- (religious OR spiritual OR cultural OR gender OR sexual) AND diversity AND (“quality of life” OR “wellbeing” [Mesh] OR “wellbeing” OR care) AND (“elderly people” OR “nursing homes” [Mesh] OR elder OR “old people”).
- A total of 377 results were obtained. After entering all the eligibility criteria: publication date from 2019 to 2023, availability of the full open text, Spanish or English language, a total of 9 studies were obtained for reading the title and abstract.

#### 2.2.6 Cochrane library

- The result of the search combination in the “Title, summary, keyword” section was:
- (religious OR spiritual OR cultural OR gender OR sexual) AND diversity AND (“quality of life” OR “well being” OR “well-being” OR care) AND (“elderly people” OR “nursing homes” OR elder OR “old people”) AND “intersectionality”
- The search was carried out in the essays section. A total of 126 trials were obtained. After entering the filters: English language; publication date: between 2019 and 2023, a total of 64 articles were obtained for reading the title and abstract.

#### 2.2.7 Scielo

- The search was carried out within “Topic” with the following combination:
- (religious OR spiritual OR cultural OR gender OR sexual) AND diversity AND (“quality of life” OR “well being” OR “well-being” OR care) AND (“elderly people” OR “nursing homes” OR elder OR “old people”) AND “intersectionality”.
- A total of 18 results were obtained. After entering all the eligibility criteria: publication date from 2019 to 2023, availability of the full open text, Spanish or English language, a total of 7 studies were obtained for reading the title and abstract.

**FIGURE 1:**
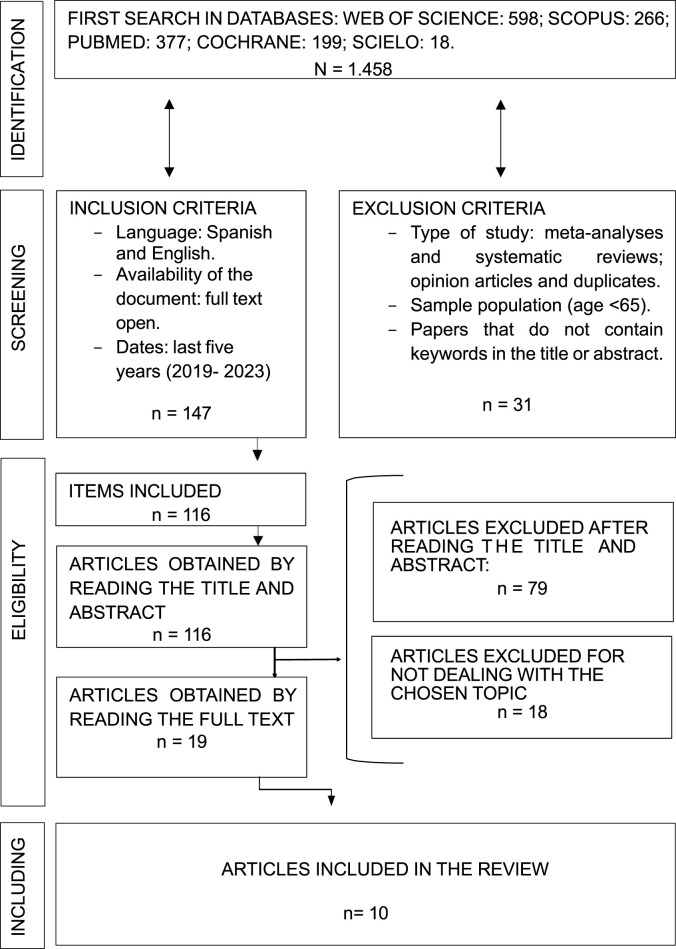
Process to determine qualifying literature to include in systematic review.

The search flowchart used during the systematic review is shown in detail below (Figure 1).

### 2.8 Methodological quality assessment

 A quality assessment of the articles included in the review was carried out by the Center for Evidence-Based Medicine (CEBM), Oxford. It is characterized by evaluating the evidence according to the thematic area or clinical scenario and the type of study that involves the clinical problem in question. It has the advantage of grading the evidence according to the best design for each clinical scenario, granting it intentionality and adding the systematic reviews (SR) in the different areas (Tables 3, 4).

**TABLE 3:** CEBM Recommendation degree and levels of evidence.

Recommendation degree	Level of evidence	Type of study
A	1a	Randomized Controlled Trial (RCT) systematic review (homogeneous among them)
	1b	Individual RCT
	1c	Clinical practice (all of them or none)
B	2a	Systematic revision/Meta-analysis of cohort studies
	2b	Cohort individual Studies (including low quality RCT)
	2c	Outcomes research. Ecological studies
	3a	Systematic review of cases-control studies
	3b	Individual study of cases and controls

Source: Adapted from “Levels of evidence and grades of recommendation” of Gorodosti (2008).

**TABLE 4:** CEBM scale for articles included in the review peer-reviewed literature.

No	Author and year of publication	Language	Level of evidence (b)	Grade of recommendation
1	Ward et al. (2019)	English	1	A
2	Villar et al. (2020)	English	2	B
3	Fredriksen-Goldsen et al. (2023)	English	1	A
4	Nelson-Becker and Thomas (2020)	English	2	B
5	Holman et al. (2020)	English	1	A
6	Willis et al. (2020)	English	1	A
7	Emlet et al. (2020)	English	1	A
8	Daley et al. (2020)	English	1	A
9	Emlet et al. (2019)	English	1	A
10	Hutchinson et al. (2022)	English	1	A

## 3 Results

### 3.1 Articles examining diversity in aging and assessing aspects of support for diverse populations in healthcare facilities and nursing homes

Of the 19 articles, based on the criteria, 10 articles were selected to be included in the analysis.

The data regarding the authors, year of publication, country, translation of the original title, type/sample and the main findings obtained from the 10 included articles are represented in a tabulated manner (Supplementary Table S5).

## 4 Discussion and conclusion

The data collected has made it possible to update the available scientific evidence on diversity and older people. It has been found that the individualized attention favors older people, but the limited research on this subject of study indicates the need for further research.

In terms of understanding the needs of older adults in relation to multicultural diversity, it can be confirmed by Hutchinson et al. (2022) that culturally and linguistically diverse older people attach great importance to the six dimensions of the Quality of Life - Aged Care Consumer (QOL-ACC). These quality-of-life dimensions refer to mobility, pain management, independence, emotional wellbeing, social connections and activities. Two other dimensions were added at the request of professionals as relevant, “identity” and “purpose,” which were found to be important for culturally and linguistically diverse older adults (CALD) but were not found to be generically the most important. Based on a study conducted by Palacios-Ceña et al. (2016), Hutchinson et al. (2022) highlight that meaningful activities are part of identity and promote a sense of belonging. Following on from the article by Hutchinson et al. (2022), two final issues are highlighted, firstly, how the global COVID-19 pandemic affected the quality of life of older people, especially their personal and social relationships as they were unable to go outdoors. Secondly, it then reaffirms the importance of identity, purpose and meaning in CALD elders and its benefit on quality of life through participation in meaningful activities.

Regarding reviewing the information on the need to include interventions on diversity care in nursing homes, it can be seen, both in the study by Ward et al. (2019) and Daley et al. (2020), that interventions on diversity care for older people are essential. The article number 1 reviews geriatric assessments in multicultural immigrant populations. The authors affirm the need for comprehensive geriatric assessments in nursing homes, which consider criteria related to multiculturalism and whose aim is to improve quality of life. To this end, a rubric for good practice is created. The article number 2 deals with LGTBI + elderly people in home care and establishes two relevant findings. Firstly, the importance of creating a structure based on the life stories and the perspective of each service user to improve their access, and the invisibility of LGTBI + older people in organizations. In turn, the same article showed that older transgender people avoid home care services for fear of rejection. Nursing homes as social and healthcare spaces must be aware of this fact, and according to Willis et al. (2020), to build accessible spaces, it is essential to establish a gender affirmative model of care.

In relation to the need for training of professionals, in article number 7 the emphasis is placed on the need for training in the field of health on gender identity diversity, avoiding cisnormativity practices. In this same area of health, it showed the disparities of people with HIV. It is evident that older homosexual and bisexual people with HIV have poorer health. There is also a depressive symptomatology, which not only affects health on a physical level, but also on a psychological level (Emlet et al., 2020). Once again, this article makes special mention of the importance of using the life histories of older people as a tool to improve the quality of life in the service, as well as the low level of training of professionals in relation to LGTBI + older people.

This same author, Emlet et al. (2019) previously conducted a study through WHOAIDS analyzing the global impact of HIV on older LGTBI + people. The study showed there is an intersectionality of stigma, affecting the person in a holistic way and how there is a growth of HIV in older people. HIV broke out in 1981, that same year Acquired Immune Deficiency Syndrome (AIDS) was recognized for the first time as a new disease (Sharp and Hahn, 2011). This means that the people of that generation who survived HIV, today could be 59 or more years old. This assumption is made with the aim of highlighting the need to work on this issue, as they are the closest generations to be potential users of nursing homes. According to Green et al. (2007) HIV in 1982 was more common in homosexual men and intravenous drug users, the first theories about its cause focused on “lifestyle” problems. This discourse persists in society and is directly related to depressive symptomatology in LGTBI + older people (Emlet et al., 2020).

Following the analysis of Holman et al. (2020), article number 5, about the need for the training in LGTBI + diversity for professionals in the care of the elderly helps to create a better climate. Therefore, this has a beneficial impact on the quality of life of users, strengthening ties and creating a healthy and safe environment. It is also evident that in organizations, in this case of a domiciliary nature, training in affective-sexual diversity is necessary. There are LGTB + older adults, especially transgender people, who avoid this type of resource for fear of rejection (Daley et at., 2020).

The study by Nelson-Becker and Thomas (2020) concludes that resilience is a fundamental factor in expanding the sense of an integrated and dignified self. The authors stress the importance of spiritual resilience and how it involves resistance through progressive self- awareness, facilitating inner transformation. In some cases, the person feels rejected by those who profess a certain religion, this can be either through their own beliefs or because of the characteristics of the person or the community. This was consolidated in the data obtained in the surveys they received. The recognition of strengths and capacities must always be included. This allows LGTBI + older people and CALD older adults to deal with marginalization. It is essential to recognize the importance of the interventions considering the life history of the service users using PCCM as a management model. This model places an emphasis on the life history of the people and the importance of their needs. As mentioned by Nelson-Becker and Thomas (2020), “people are resilient because they often have few other options except to despair and isolate themselves. Their tenacity to reclaim a personal sense of spirituality and find meaning in adverse situations is a testament to this resilience”.

It is vitally important to carry out interventions where older people are the center of attention. For this reason, the PCCM was established as a management model for the centers. Its realization and execution from intersectionality is of vital importance. The interventions must be carried out in the nursing homes using the PCCM, including the staff in this change so that they feel part of it. It is essential for the workers to be trained in diversity, thus reducing age discrimination, stigmas associated with religion and ethnicity and creating spaces of equality and support for emotional-sexual diversity. It should include the establishment of diverse and inclusive policies, staff diversity and sensitivity, personalized care plans, addressing it from community and family participation.

### 4.1 Detailed implementation strategies are crucial for the effective application of the person-centered care model (PCCM) in nursing homes

#### 4.1.1 Implementation in real time

Implementing PCCM in real-time within nursing homes involves several steps to ensure it is practical and beneficial for residents:

a) Interdisciplinary Team Approach: Nursing homes should establish an interdisciplinary team that includes a clinical psychologist, behavioral specialist, social worker, and counselor. This team should meet with each new resident upon their arrival to develop a personalized care plan. This initial assessment should be comprehensive, considering the resident’s physical health, psychological state, social needs, and personal preferences.
b) Regular Review and Adaptation: The care plan should not be static. Regular meetings (e.g., quarterly) should be scheduled to review and adapt the care plan based on the resident’s evolving needs and preferences.
c) Training and Education: Continuous education and training for all staff members on PCCM principles and practices are essential. This training should emphasize the importance of empathy, active listening, and personalized care strategies.

#### 4.1.2 Creating the care plan

The creation of the care plan should be a collaborative process involving multiple stakeholders:

a) Clinical Psychologist: To assess and address mental health needs, providing strategies to manage anxiety, depression, and other psychological issues.
b) Behavioral Specialist: To design behavior management plans and interventions tailored to the resident’s specific needs.
c) Social Worker: To identify and address social needs, including family dynamics, social interactions, and community engagement.
d) Counselor: To provide emotional support and counseling, helping residents cope with the transition and any personal issues.

#### 4.1.3 Reality of personalized care in large facilities

In large nursing homes with over 100 residents, implementing and maintaining individualized care plans can indeed be challenging. However, several strategies can mitigate these challenges:

a) Delegation and Team Structure: Assigning specific residents to designated team members who are responsible for their care plan ensures accountability and continuity. This can be managed through smaller sub-teams within the larger interdisciplinary team.
b) Technology Utilization: Implementing electronic health records (EHR) and specialized software to track care plans, monitor updates, and ensure that all staff have access to up-to-date information about each resident.
c) Incremental Implementation: Start with a pilot program for a smaller group of residents, refining the process before scaling it up to the entire facility.

#### 4.1.4 Addressing temporary and high turnover in staff

The presence of temporary staff and high turnover rates present significant barriers to consistent and personalized care:

a) Standardized Procedures: Develop detailed, standardized procedures and protocols that temporary staff can easily follow. This includes thorough documentation in the EHR that allows new staff to quickly understand each resident’s needs and preferences.
b) Orientation and Training for Temporary Staff: Implement a robust orientation program for temporary staff, focusing on the core principles of PCCM and the specific practices of the nursing home.
c) Retention Strategies: Investing in strategies to improve staff retention, such as competitive salaries, benefits, professional development opportunities, and a supportive work environment, is critical for maintaining a stable and consistent caregiving team.

#### 4.1.5 Need for systemic reform

The current climate in many nursing homes necessitates significant reform to support such a paradigm shift:

a) Policy Advocacy: Advocate for policies that support better staffing ratios, increased funding for staff training, and incentives for nursing homes to adopt PCCM.
b) Community and Family Engagement: Engage families and the broader community in supporting nursing homes, whether through volunteer programs, donations, or advocacy efforts.
c) Continuous Improvement: Establish a culture of continuous improvement within nursing homes, where feedback from residents, families, and staff is actively sought and used to make ongoing enhancements to care practices.

In conclusion, while implementing PCCM in nursing homes presents challenges, especially in large facilities or those with high staff turnover, these challenges are not insurmountable. With a strategic approach, adequate resources, and a commitment to continuous improvement, it is possible to create a more inclusive, empathetic, and effective care environment for older adults.

### 4.2 Study limitations

This review has several limitations. Firstly, methodological in nature. There were many articles that could not be accessed due to their “restricted access” nature, so much information about the subject of the study has already been lost along the way. Secondly, the scarcity of published studies that evaluate results in terms of attitudes to diversity in older people, both in nursing homes and in potential users of the services.

A more exhaustive study of the literature is needed, with free access to the available literature to be able to reflect more fully the results of the articles that deal with the subject. This future line of research in turn requires the definition of a concrete and specific protocol in terms of the tools used, the number and design of the sessions and, in short, the application of an intervention in which the maximum possible number of variables that influence this type of study are controlled.

Finally, it is of interest to continue with future lines of research regarding the attitude towards diversity, along with studying the effects of the changes using the PCCM and its implications on the quality of life for older people who use the services.

## Data Availability

The raw data supporting the conclusions of this article will be made available by the authors, without undue reservation.
