# Supplementary material for: Systematic review in relation to support of diversity in nursing homes
Source: Front Aging. 2024 Sep 24;5:1389610. doi: 10.3389/fragi.2024.1389610 (PMC11458523; doi:10.3389/fragi.2024.1389610)
Supplement: Supplementary file 1 [file Table1.docx]

**Table 5**

Summary of studies included in the review and major findings related to diversity in nursing homes

| **No.** | **1** |
| --- | --- |
| **Key aspects** | **Old people / multicultural / diversity / geriatric** |
| Country | USA |
| Title | Geriatric evaluation in multicultural immigrant populations. |
| Type/Sample | Multiculturalism in older adults. |
| Goals | Propose a comprehensive geriatric evaluation that includes ethnography. |
| Main findings | - There is a growing need for a more culturally sensitive comprehensive geriatric assessment to adequately address the complex social and cultural needs of immigrant older adults to improve clients' quality of life. - Creating a rubric for best practices in a multicultural assessment of geriatric immigrants, identifies gaps in existing approaches. |
| **No.** | **2** |
| **Key aspects** | **Long-term facilities / elder / LGTBI+ / SEXUAL NEEDS** |
| Country | Spain |
| Title | 'I do it my way': perceptions of care staff in long-term care facilities about residents' sexual needs and suggestions to improve their management. |
| Type/Sample | Randomized controlled trial.   - 2115 labor personnel. - In 152 Spanish residences. - 161 professionals who worked in management positions (7.60%). - 614 technical staff (29%) and 1,340 care assistants (63.40%). - The mean size of facilities was 86.56 residents (*SD* = 45.71). Most were public institutions (42.1%), while 38.8% were private for-profit institutions. The remainder (19.1%) were private not-for-profit institutions. |
| Goals | To explore the extent to which staff perceive that older residents in long-term care facilities (LTCFs) still have sexual needs, and how they think care in relation to sexual problems could be improved. |
| Main findings | - Staff working in LTCF long-term care facilities do not see sexual needs as something still present in many (or even some) older people living in LTCF. - This inability to conceive of sexual needs as basic, universal needs could explain why (a) sexuality does not appear to be a priority in LTCFs and (b) explicit policies to manage sexual needs and guarantee rights are often absent. - The application of a person-centered care model, which considers the rights of residents and seeks to adapt care according to residents' life stories and priorities, has been proposed as a necessary step. - The average score obtained in response to the question about how many older people living in LTCFs have sexual needs was 2.93 (SD = 0.82, range 1–5). While 26.2% of the participants thought that none or just a few residents have sexual needs, 19.1% attributed such needs to most or all residents. |
| **No.** | **3** |
| **Key aspects** | **SEXUAL AND GENDER MINORITY / OLDER DULTS** |
| Country | USA |
| Title | Design and development of the first randomized controlled trial of an intervention (“Innovations in Dementia Empowerment and Action” [IDEA]) for sexual and gender minority older adults living with dementia and caregivers. |
| Type/Sample | Randomized controlled trial.   - 300 LGTB+ older adults with dementia. - ≥ 60 years. |
| Goals | Describe the design of the first randomized controlled trial (RCT) testing a culturally sensitive cognitive behavioral and empowerment intervention, Innovations in Dementia Empowerment and Action (IDEA), developed to address the unique needs of SGM older adults (“sexual and gender minority” [SGM]) living with dementia and their care partners. |
| Main findings | - Compared to their heterosexual age peers, sexual and gender minority (SGM) older adults show worse health outcomes and risk factors. - IDEA tests culturally tailored intervention for older SGM adults with dementia and caregivers. - IDEA incorporates empowerment practices: commitment, effectiveness, mobilization of support. - The personalized SGM intervention addresses SGM stigma, identity management, bias, and trauma. - To detect a small effect (*d* = 0.25), which is greater than a minimal clinically important difference level [[39](https://www.ncbi.nlm.nih.gov/pmc/articles/PMC10639124/#R39)], assuming statistical power of 0.8 and significance level of 0.05, the estimated sample size was 52 dyads in each group (104 dyads total). Adjusting the sample size up to account for the projected 25% attrition over two years indicated a total sample to recruit of 150 dyads. |
| **No.** | **4** |
| **Key aspects** | **Religious / Spiritual / LGTBI+ / RACIALIZED OLDER PEOPLE** |
| Country | USA |
| Title | Religious/spiritual struggles and spiritual resilience in marginalized older adults. |
| Type/Sample | Mixed methods study, both at a descriptive and narrative level: interviews and data analysis.   - 143 people. |
| Goals | Provide a foundation for the discussion of spiritual resilience in older people through examples from two different community studies: 55 older LGBTI+ dyads in various nations, and 75 racialized older people. |
| Main findings | The properties of resilience across life stories in aging can be expanded to include the sense of an integrated and dignified self and the recognition of the strengths and capabilities that enable older LGBTI+ people and older adults from ethnic minority backgrounds. and religious people deal with marginalization and, in some cases, overcome it.   - As the data from the couple studies reported here demonstrate, some LGBQ people see themselves as excluded from mainstream religious faiths. |
| **No.** | **5** |
| **Key aspects** | **LGTBI+ / OLDER ADULTS / DIVERSITY TRAINING / NURSING HOME** |
| Country | USA |
| Title | Creating supportive environments for LGBTI+ older adults: an evaluation of the effectiveness of staff training in a senior center. |
| Type/Sample | Randomized controlled trial.   - 904 people main study. - N=43 specific study. |
| Goals | Evidence the effectiveness of training staff in gerontological centers on how to create a more favorable living environment for LGBTI+ elderly people. |
| Main findings | - This study provides evidence that a 4-hour face-to-face LGBT diversity training can help create a more favorable housing climate for LGBT elders by increasing staff knowledge and competence, improving their quality of life. - This educational staff training model offers a framework to strengthen the optimal aging of LGBT seniors through creating supportive living environments and, ultimately, a safe and healthy environment for LGBT seniors. - Results from independent sample *t*-tests (see [Table 2](https://www.ncbi.nlm.nih.gov/pmc/articles/PMC8015408/table/T2/)) showed that post-training intervention perceived preparedness scores differed by staff designation (*t* = 2.39, *p* = .045), whereby those in facilities services reported higher perceived preparedness than healthcare service staff. LGBT content knowledge prior to the training intervention was higher among those with at least some college education relative to those without (*t* = −3.92, *p* = .001). Preintervention LGBT content knowledge and post-intervention supportive attitudes also varied by religion. Specifically, compared to those reporting Agnostic and “Other” religions, those who reported being Christian had lower post-intervention content knowledge (*t* = −2.20, *p* = .035) and lower post-intervention supportive attitudes (*t* = −2.32, *p* = .027). Given these findings, we include these variables as covariates in our repeated measures ANCOVA. |
| **No.** | **6** |
| **Key aspects** | **OLDER PEOPLE / TRANSGENDER / STEREOTYPES / HEALTH CARE SERVICES** |
| Country | United Kingdom |
| Title | Reluctant educators and self-advocates: Experiences of older transgender adults in health care services and professionals seeking gender-affirming services. |
| Type/Sample | Qualitative study through the structured method approach.   - 22 people. |
| Goals | - Examine the supporting and obstructive points of interaction with health professionals. - Identify key learning messages to improve trans-related healthcare from the perspective of adults who identify as trans people in adulthood. |
| Main findings | - Clinical pressures to conform to gender stereotypes. - For adults transitioning into their 50s and 60s, there is an underlying chronological imperative to 'run out of time' to complete this life-changing journey. - Messages from this study speak to the importance of improving health‐care professionals’ knowledge of gender identity diversity across the life course and making changes at a systemic level in redressing cisnormative systems and practices. |
| **No.** | **7** |
| **Key aspects** | **HEALTH DISPARITIES / LGTBI+ / OLDER ADULTS / HIV** |
| Country | USA |
| Title | Considering human immunodeficiency virus (HIV) health disparities: Risk and protective factors among older gay and bisexual men. |
| Type/Sample | Randomized controlled trial.   - 1344 older (50+) gay/bisexual men with and without (n = 973) HIV. |
| Goals | To examine whether disparities exist in poor health and depressive symptomatology among older (50+) gay/bisexual men with (n = 371) and without (n = 973) HIV. If so, what risk/promotion factors explain these disparities? |
| Main findings | - People with HIV reported poorer health and more depressive symptomatology due to decreased income, resilience and social support, and greater lifetime victimization. - Older gay/bisexual men living with HIV infection are at increased risk for physical and mental health problems. - The direction of indirect effect via LGBT community engagement was different from the other factors stated above, such that the risk of poorer general health was lower for those with HIV (as indicated by the negative coefficient, −0.02) on account of their level of LGBT community engagement being higher than those without HIV. - Those with HIV were to have lower scores of depressive symptomatology (coefficient = −0.09) on the account of their level of LGBT community engagement being higher than those without HIV. |
| **No.** | **8** |
| **Key aspects** | **LGTBI+ / HOME CARE SERVICES / TRAINING AND EDUCATION** |
| Country | USA |
| Title | A framework to improve access to equitable home care for LGBTI+ (2SLGBTQ+) senior communities |
| Type/Sample | Mixed methods study, both at a descriptive and narrative level: surveys, individual interviews and focus groups.   - 621 people. |
| Goals | - Create a framework for access and equity, using the stories and perspectives of 2SLGBTQ+ participants to improve access to home care. - Underline the relevance and effectiveness of said structure as a tool to support systematic organizational assessment, evaluation and implementation of access and equity strategies. |
| Main findings | - Invisibility of 2SLGBTQ+ in home care organizations. - Lack of training and education on 2SLGBTQ+ among home care service providers. - Avoidance of home care services by trans people. - The Home Care Access and Equity Framework contributes to scholarship on 2SLGBTQ+ access and equity, providing a framework and process for organizational EDI change strategies within home care settings specifically and in health and social care service organizations generally. |
| **No.** | **9** |
| **Key aspects** | **HIV / SEXUAL AND GENDER MINORITY / OLDER ADULTS** |
| Country | USA |
| Title | The global impact of HIV on sexual and gender minority older adults: Challenges, progress, and future directions. |
| Type/Sample | Data analysis through UNAIDS. |
| Goals | Provide an overview of how HIV is affecting older adults globally, with a focus on sexual and gender minority older adults. |
| Main findings | - Intersectionality of stigma. - Increasing prevalence of HIV among older people. - Need for improved and acceptable testing and treatment options. - Increase in multimorbidity. - An estimated 100,000 people aged 50 and over in low- and middle-income countries become infected with HIV each year. |
| **No.** | **10** |
| **Key aspects** | **QUALITY OF LIFE / LGTBI+ / OLDER ADULTS / NURSING HOMES** |
| Country | Switzerland |
| Title | What dimensions of quality of life are most important for older adults from culturally and linguistically diverse backgrounds receiving aged care services? An exploratory study |
| Type/Sample | Mixed methods study at a descriptive and narrative level: focus groups, semi-structured interviews, and surveys.   - 102 older adults culturally and linguistically diverse (CALD) |
| Goals | Identify which dimensions of quality of life were most important for older adults (CALD) |
| Main findings | The importance of the six dimensions of the QOL-ACC (mobility, pain management, independence, emotional well-being, social connections and activities) is confirmed for this sample of CALD older adults living in residential aged care, as well as in the community.   - Three organisations agreed to participate in focus groups. Focus group 1 was with an ethno-specific aged care provider (‘ethno-specific’, n = 2), focus group 2 was with a provider that had an ethno-specific unit at one of its facilities but otherwise offered mainstream aged care services (‘hybrid’, n = 3), and focus group 3 was with a provider that only offered mainstream services (‘mainstream’, n = 4). The focus groups were conducted by a facilitator and the first author. |
